# Glomerular Deposition of Nephritis-Associated Plasmin Receptor (NAPlr) and Related Plasmin Activity: Key Diagnostic Biomarkers of Bacterial Infection-related Glomerulonephritis

**DOI:** 10.3390/ijms21072595

**Published:** 2020-04-08

**Authors:** Takahiro Uchida, Takashi Oda

**Affiliations:** Kidney Disease Center, Department of Nephrology and Blood Purification, Tokyo Medical University Hachioji Medical Center, Hachioji, Tokyo 193-0998, Japan; takashio@tokyo-med.ac.jp

**Keywords:** poststreptococcal acute glomerulonephritis, infection-related glomerulonephritis, nephritis-associated plasmin receptor, plasmin

## Abstract

It is widely known that glomerulonephritis (GN) often develops after the curing of an infection, a typical example of which is GN in children following streptococcal infections (poststreptococcal acute glomerulonephritis; PSAGN). On the other hand, the term “infection-related glomerulonephritis (IRGN)” has recently been proposed, because infections are usually ongoing at the time of GN onset in adult patients, particularly in older patients with comorbidities. However, there has been no specific diagnostic biomarker for IRGN, and diagnosis is based on the collection of several clinical and pathological findings and the exclusion of differential diagnoses. Nephritis-associated plasmin receptor (NAPlr) was originally isolated from the cytoplasmic fraction of group A streptococcus as a candidate nephritogenic protein for PSAGN and was found to be the same molecule as streptococcal glyceraldehyde-3-phosphate dehydrogenase and plasmin receptor. NAPlr deposition and related plasmin activity were observed with a similar distribution pattern in the glomeruli of patients with PSAGN. However, glomerular NAPlr deposition and plasmin activity could be observed not only in patients with PSAGN but also in patients with other glomerular diseases, in whom a preceding streptococcal infection was suggested. Furthermore, such glomerular staining patterns have been demonstrated in patients with IRGN induced by bacteria other than streptococci. This review discusses the recent advances in our understanding of the pathogenesis of bacterial IRGN, which is characterized by NAPlr and plasmin as key biomarkers.

## 1. Introduction

A wide variety of bacterial infection-related renal diseases are known, among which the most common is acute kidney injury (AKI) [1], which occurs as part of multiple organ failure. Changes in hemodynamics and cytokine expression are thought to be involved in the pathogenesis of AKI.

Bacterial infections also cause renal injury, partly through immune mechanisms. For example, glomerulonephritis (GN) can develop following streptococcal upper respiratory tract or skin infections with a latent period of approximately 10 days. As streptococcal infections are usually cured when GN is diagnosed and there is a distinct infection-free latent period, the GN has been referred to as poststreptococcal acute glomerulonephritis (PSAGN) [2,3,4].

In previous years, most cases of AGN were PSAGN in children; however, probably owing to the improvement of living environments and the adequate usage of antibiotics, the incidence of PSAGN has been decreasing, particularly in developed countries [4]. Whereas PSAGN is still the most common cause of pediatric AGN, adult AGN cases have been increasing, and those associated with non-streptococcal infections, particularly infections by *Staphylococcus aureus*, are now as common as PSAGN [5]. Thus, a major shift in the epidemiology of AGN has occurred. In Japan, cases of PSAGN, which accounted for more than two-thirds of AGN cases in the 1970s, decreased to about 30% after the 1980s, whereas AGN cases associated with *S. aureus* infections reached 30% in the 1990s [6].

Furthermore, in adult AGN patients, the infection is usually still present at the time when GN is diagnosed. Based on these backgrounds, instead of “postinfectious AGN”, the disease concept of infection-related glomerulonephritis (IRGN) has recently been proposed [5]. Notably, whereas in most patients, PSAGN resolves without any specific therapy, the prognosis of patients with IRGN is poor, and older patients, particularly those with an immunocompromised background, such as diabetes mellitus, malignancies, or alcoholism, are reported to be at high risk [7]. Controlling the underlying infection and managing complications are essential for the treatment of IRGN, and immunosuppressive therapy is generally not recommended. However, the prompt diagnosis of IRGN is often difficult because specific diagnostic biomarkers have not yet been identified.

We herein present an overview of our recent understanding of the pathogenesis of bacterial IRGN. Accumulated data suggest that the disease concept of bacterial IRGN can be further expanded, and glomerular deposition of nephritis-associated plasmin receptor (NAPlr), originally considered to be a candidate nephritogenic protein for PSAGN [8] and related plasmin activity [9], can be used as general diagnostic biomarkers of bacterial IRGN. Although infections of various viruses, mycobacteria, fungi, or protozoa are also known to cause IRGN [1], they are not within the scope of this article.

## 2. NAPlr and Plasmin Activity in Glomeruli as Biomarkers of PSAGN

NAPlr is a 43-kDa protein that was originally isolated from the cytoplasmic fraction of group A streptococcus as a candidate nephritogenic protein for PSAGN [8]. Glomerular NAPlr deposition is detected by immunostaining, and is frequently observed in the early phase of PSAGN; all patients within 2 weeks of disease onset have been reported to show NAPlr deposition [2].

NAPlr was also found to be the same molecule as streptococcal glyceraldehyde-3-phosphate dehydrogenase (GAPDH) [8]. Although GAPDH is a well-known housekeeping gene, it also has pleiotropic functions, such as energy production (glycolysis), regulation of gene expression, and autophagy [10]. In addition, GAPDH from some bacteria, including streptococci, has been shown to have plasmin-binding activity [11,12].

NAPlr binds plasmin and maintains plasmin activity by protecting it from its physiological inhibitors. Plasmin activity can be detected by *in situ* zymography using a plasmin-sensitive synthetic substrate, which is resistant to the addition of α_2_-antiplasmin but is completely abrogated by aprotinin, a serine protease inhibitor [9]. Plasmin is considered to cause glomerular damage directly by degrading extracellular matrix proteins and indirectly by activating pro–matrix metalloproteases. Additionally, plasmin can exert proinflammatory function by activating and accumulating inflammatory cells.

NAPlr is also known to convert complement component C3 to C3b, indicating its involvement in the activation of the alternative complement pathway [8]. However, it should be noted that NAPlr deposition is observed mainly in glomerular neutrophils, mesangial cells, and endothelial cells, and its distribution in glomeruli is different from that of C3 and IgG, which are considered to localize within the subepithelial hump [13]. In this regard, NAPlr, which also contains a urokinase-type plasminogen activator receptor (uPAR)-binding site [11], may bind with uPAR expressed on neutrophils, thereby inducing prominent endocapillary inflammation in early phase PSAGN, or NAPlr may be phagocytosed by neutrophils as exogenous material. Thus, glomerular damage from the disease may initially occur in the inner side of the glomerular capillary walls by NAPlr deposition, rather than subepithelial immune complexes.

**Figure 1:**
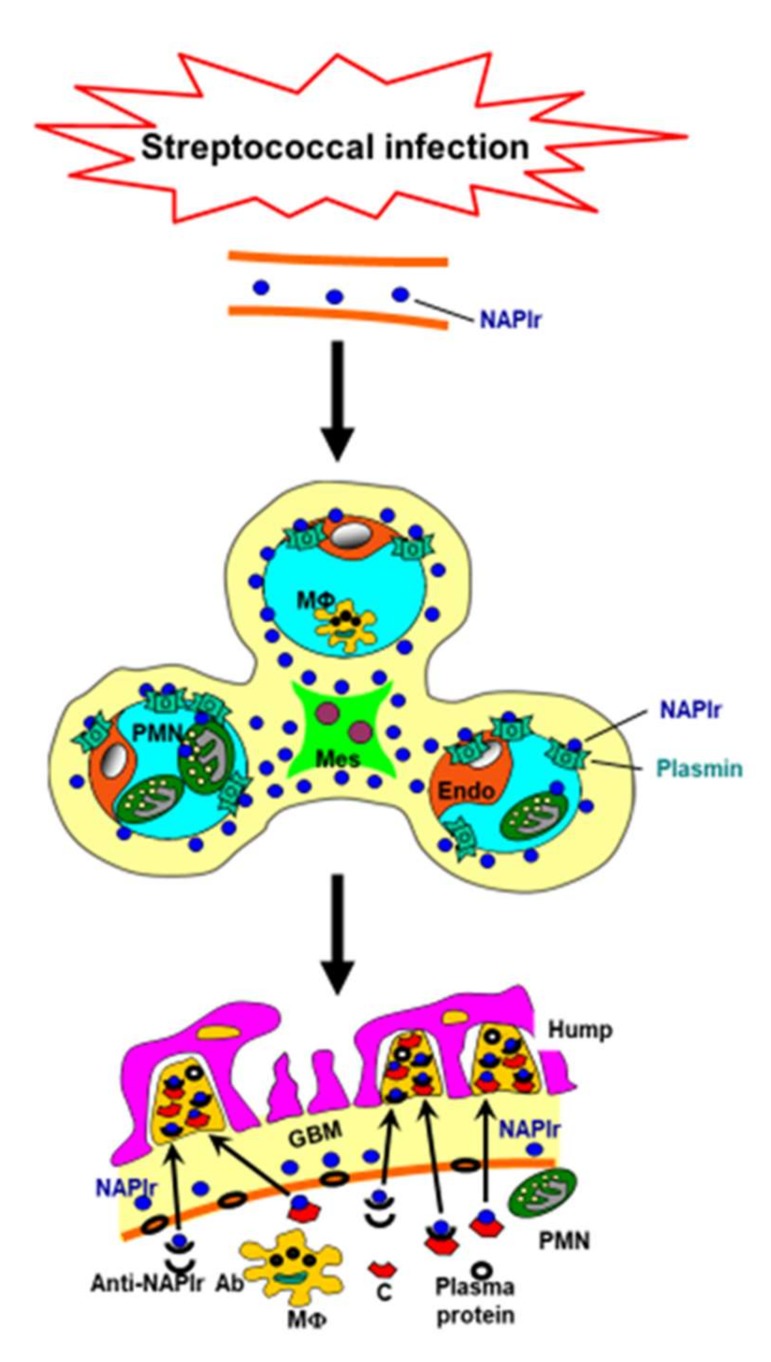
Putative mechanism for the development of poststreptococcal acute glomerulonephritis. Streptococcal infection induces the release of nephritogenic proteins, such as nephritis-associated plasmin receptor (NAPlr), into the circulation. Circulating NAPlr accumulates on the inner side of the glomerular capillary walls, and then traps and maintains the activity of plasmin, which induces glomerular damage by the degradation of extracellular matrix proteins or by activating and accumulating inflammatory cells. Thereafter, immune complexes, formed either *in situ* or in the circulation, pass through the altered glomerular basement membrane (GBM). Accumulation of immune complexes, complements, and plasma proteins forms “humps” on the outer side of the glomerular capillary walls. This scheme is based on the figure from Oda et al. [2]. Ab: antibody; C: complement; Endo: endothelial cell; Mes: mesangial cell; MΦ: macrophage; PMN: polymorphonuclear cell.

Streptococcal pyrogenic exotoxin B (SPEB), which is another potential nephritogenic protein of PSAGN with cationic character, has been considered to pass through the glomerular basement membrane and be deposited in the subepithelial area [14]. However, a subsequent study showed that the glomerular distribution of NAPlr and SPEB were essentially similar and that NAPlr staining was dominant [13]. Importantly, as with NAPlr, SPEB has plasmin-binding activity [5]. Thus, it is possible that these 2 (or more) proteins are cooperatively involved in the disease pathogenesis of PSAGN. Figure 1 shows a scheme of the mechanisms involved in the development of PSAGN.

Some streptococcal strains have been isolated from PSAGN patients, and such strains have been considered to be “nephritogenic”. However, NAPlr, as well as SPEB, have been found in virtually all streptococcal strains [14]. In addition, gene sequences of NAPlr were highly conserved and its protein expression levels were similar between various streptococcal strains [15]. Therefore, it is reasonable to think that any strains expressing NAPlr can be nephritogenic. Another possibility also remains that although NAPlr (and SPEB) are essential molecules, other factors, from both bacteria and the hosts, play important roles in the onset/progression of PSAGN.

There have been several reports showing the occurrence of IRGN in renal transplant recipients, suggesting that IRGN may be a cause of renal allograft injury [16,17]. We have recently encountered a renal transplant recipient who developed PSAGN; notably, NAPlr deposition and plasmin activity were observed in the glomeruli of the patient’s transplanted kidney (manuscript in preparation).

## 3. Streptococcal Infection-related Nephritis (SIRN): Glomerular Diseases with NAPlr Deposition and Related Plasmin Activity Induced by Streptococcal Infection

The unique glomerular staining patterns of NAPlr and plasmin activity were found not only in patients with PSAGN but also in some patients with other glomerular diseases, such as C3 glomerulopathy [18,19], membranoproliferative glomerulonephritis (MPGN) type I [20,21], antineutrophil cytoplasmic antibody (ANCA)-associated vasculitis (both ANCA positive [22] and negative [23]), and IgA vasculitis [24], in which a preceding streptococcal infection is suggested by serological markers, and these cases are referred to as SIRN [2,25]. Although prominent endocapillary proliferation is a common histological feature, the differences in immune responses of the affected hosts may affect the specific histology. In addition, there have been some cases in which patients had a preceding streptococcal infection that initially occurred as PSAGN but later developed into C3 glomerulopathy [26]. It has also been reported that C3 nephritic factor activity is transiently observed in some PSAGN patients during the acute phase of the disease [27]. Thus, the disease concept of SIRN remains to be established, and there are no specific criteria differentiating patients with SIRN from those with the above glomerular diseases. However, it should be noted that streptococcal infections are associated with several forms of GN and that NAPlr and plasmin activity may be biomarkers of these diseases.

## 4. Glomerular NAPlr Deposition and Plasmin Activity as Candidates of General Biomarkers of Bacterial IRGN

The diagnosis of IRGN is made based on a combination of clinical and pathological findings. Nasr et al. [5] proposed the diagnostic criteria for IRGN as follows, in which at least 3 of the 5 items are required for a positive diagnosis: (1) clinical or laboratory evidence of infection preceding or at the onset of GN, (2) decrease in serum complement levels, (3) endocapillary proliferative and exudative glomerulonephritis, (4) C3-dominant or codominant glomerular immunofluorescence staining, and (5) hump-shaped subepithelial deposits on electron microscopy. The most common pathogen causing bacterial IRGN is staphylococcus, followed by streptococcus and Gram-negative bacteria. Infection sites are diverse, including the upper respiratory tract, skin, lung, and urinary tract, and the identification of foci is sometimes difficult. In addition, disease-specific diagnostic biomarkers have not been identified to date, and there are usually several differential diagnoses that show confounding clinical or pathological characteristics. Therefore, the prompt and accurate diagnosis of IRGN is often difficult.

Interestingly, glomerular NAPlr deposition and plasmin activity have recently been demonstrated in patients with IRGN induced by some bacterial strains, such as *Streptococcus pneumoniae* [28], *Aggregatibacter actinomycetemcomitans* (a Gram-negative coccobacillus that sometimes causes periodontal disease and infectious endocarditis) [29], *Mycoplasma pneumoniae* [30], or *S. aureus* (both methicillin-sensitive and -resistant strains; unpublished observations). The sequences of *S. pneumoniae* GAPDH share high identity with NAPlr, and the C-terminal sequences of *S. pneumoniae* GAPDH, which are most likely to be associated with the plasmin-binding activity, are completely identical to those of streptococcal GAPDH [28]. *M. pneumoniae* GAPDH has been shown to not only have cross-immunoreactivity to the anti-NAPlr antibody but also to have a plasmin-binding function. In addition, *M. pneumoniae* GAPDH has been reported to bind to plasminogen and convert it to plasmin [31]. As shown in Table 1, sequences of GAPDH from *A. actinomycetemcomitans* and *S. aureus* also show high similarity to that of NAPlr at the amino acid level, and *S. aureus* GAPDH has been reported to bind to enzymatically active plasmin [32]. As stated above, GAPDH sequences are highly preserved between streptococcal species [15], and it is, therefore, reasonable that GAPDH from these bacteria have plasmin-binding ability and that its deposition in glomeruli is detected by the anti-NAPlr antibody. However, it should be noted that although the glomerular staining pattern of NAPlr is essentially the same among patients with IRGN, the staining intensity in patients with non-streptococcal IRGN is generally weaker than in those with PSAGN. In this regard, the timing of when a renal biopsy is performed might affect the staining intensity; it could be possible that, in patients with non-streptococcal IRGN, performing renal biopsy during the acute phase is often avoided because of comorbidities or ongoing infection. Another possibility is that positive immunostaining of NAPlr in patients with non-streptococcal IRGN is caused by cross-immunoreactivity to the anti-NAPlr antibody, and is, therefore, weaker than that in patients with PSAGN.

On the other hand, even if the overall amino acid sequence similarity is not very high, it is possible that some types of bacterial GAPDH, which have a similar steric structure at the antibody-binding site, show cross-immunoreactivity to the anti-NAPlr antibody. Indeed, positive staining for NAPlr and plasmin activity has been used as a marker of IGRN in patients with various forms of glomerulonephritis, such as proliferative glomerulonephritis with monoclonal immunoglobulin G deposits [33] and eosinophilic proliferative glomerulonephritis [34], even if neither the pathogens nor the infection sites could be identified. As the binding site of the anti-NAPlr antibody and that of plasmin in the GAPDH sequence would be different, the GAPDH of some bacteria are expected to bind with plasmin but not react with the anti-NAPlr antibody. In this regard, whether there are IRGN cases in which glomerular plasmin activity is observed without immunoreactivity to the anti-NAPlr antibody needs to be investigated in the future.

**Figure 2:**
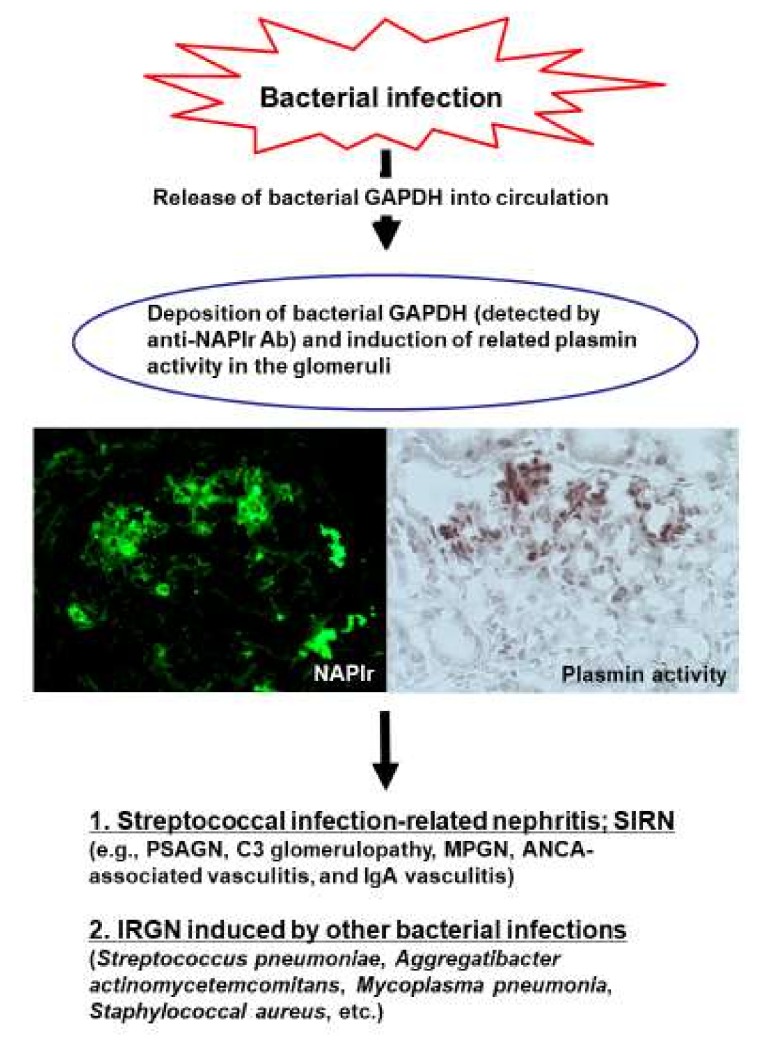
Possible scheme for the pathogenic mechanisms of bacterial infection-related glomerulonephritis (IRGN), focusing on the glomerular deposition of nephritis-associated plasmin receptor (NAPlr) and related plasmin activity. Ab: antibody; ANCA: antineutrophil cytoplasmic antibody; GAPDH: glyceraldehyde-3-phosphate dehydrogenase; MPGN: membranoproliferative glomerulonephritis; PSAGN: poststreptococcal acute glomerulonephritis.

Collectively, GAPDH from various bacteria appear to react with the anti-NAPlr antibody and to have plasmin-binding ability, and positivity of the anti-NAPlr antibody and plasmin activity in glomeruli may act as both diagnostic and pathogenetic biomarkers of bacterial IRGN in general. Putative pathogenic mechanisms of IRGN, focusing on NAPlr and plasmin activity as biomarkers, are depicted in Figure 2.

## 5. NAPlr and Plasmin Activity in Extraglomerular Regions

In the previous study, NAPlr deposition has been observed almost exclusively in the glomeruli of IRGN patients, and the question hence arises as to whether NAPlr deposition is truly limited to the glomeruli. Interestingly, a case of PSAGN complicated by acute interstitial nephritis, in which positive SPEB immunostaining was observed in the interstitium as well as in the glomeruli, has been reported [35]. Although acute tubulointerstitial nephritis after streptococcal infection without obvious GN is rare, such a case has indeed been reported, in which SPEB immunostaining was positive in the affected area [36]. In the former case report [35], the authors also performed immunofluorescence staining of NAPlr using a commercially available antibody but failed to detect its deposition. In this regard, however, it should be noted that immunostaining results can vary depending on the antibodies used and the staining conditions [2]. Thus, whether NAPlr deposition occurs in the tubulointerstitial area or not should be examined more carefully using different antibodies and staining conditions. NAPlr immunofluorescence staining using an original antibody (and *in situ* zymography for plasmin activity) can be performed at the laboratory of Dr. Takashi Oda (Kidney Disease Center, Department of Nephrology and Blood Purification, Tokyo Medical University Hachioji Medical Center; takashio@tokyo-med.ac.jp).

Tubulointerstitial plasmin activity could be found in patients with various renal diseases unrelated to bacterial infection [9,37]. However, NAPlr deposition could not be observed, and plasmin activity was almost exclusively limited to the tubulointerstitial area in the renal tissues of these patients. Although definitive causative roles remain to be solved, this plasmin activity in the tubulointerstitial area may be involved in renal tubulointerstitial inflammation and fibrosis, because plasmin is supposed to induce the infiltration and activation of inflammatory cells and to induce fibrogenesis. Indeed, tubulointerstitial plasmin activity was associated with the degree of tubulointerstitial change, global glomerulosclerosis rate, and estimated glomerular filtration rate [37]. Data regarding tubulointerstitial plasmin activity in patients with IRGN are scarce, and, hence, further accumulation of cases is needed to investigate this matter in more detail.

PSAGN patients rarely show alveolar hemorrhage, and immune complex deposition is suggested in the pathogenesis of alveolar hemorrhage [38]. Therefore, another important issue that remains to be investigated is whether or not NAPlr deposition and related plasmin activity are observed in the lung tissue of patients with PSAGN complicated by alveolar hemorrhage. In this regard, an interesting case of IRGN, in which the causative pathogen was not detected but NAPlr deposition and plasmin activity were observed not only in the glomeruli but also in the renal tubulointerstitial area and pulmonary arteries, has recently been reported [34].

## 6. Concluding Remarks

NAPlr, isolated from the cytoplasmic fraction of group A streptococcus, has been shown to trap plasmin and maintain its activity and was originally considered as a nephritogenic protein for PSAGN. Indeed, NAPlr deposition and related plasmin activity have been observed to have an almost identical distribution in the glomeruli of early phase PSAGN patients at a high frequency. The interactions among NAPlr, plasmin activity, and SPEB and the association between these elements and complements or immune complexes, both *in vitro* and *in vivo*, should be investigated in future studies.

Some patients with other glomerular diseases, in whom a preceding streptococcal infection is clinically suggested, were found to also show glomerular NAPlr deposition and plasmin activity, and hence, these cases can be referred to as SIRN. Furthermore, such glomerular-staining patterns of NAPlr and plasmin activity are found in some patients with IRGN induced by other bacteria. The amino acid sequence of GAPDH from some types of bacteria show high similarity to the sequence of NAPlr and these bacterial GAPDH molecules appear to have a plasmin-binding ability. Even if the overall similarity is not so high, it is possible that some bacterial GAPDH molecules, which have a similar steric structure at the antibody-binding site, show cross-immunoreactivity to the anti-NAPlr antibody.

It has become evident that bacterial infections are more deeply involved in various renal diseases, including IRGN, than we previously considered. Although the development of noninvasive techniques to detect infections with high sensitivity and high specificity is undoubtedly crucial, the identification of bacterial proteins associated with the pathogenesis of IRGN is also an important ongoing effort. Thus, future studies evaluating the possibility of NAPlr and plasmin activity as common diagnostic biomarkers of bacterial IRGN are anticipated.

## Figures and Tables

**Table 1:** Identity and similarity of bacterial GAPDH and streptococcal GAPDH (nephritis-associated plasmin receptor; NAPlr).

Pathogen	Nucleotide		Amino acid	
	Identity	Similarity	Identity	Similarity
*Aggregatibacter actinomycetemcomitans*	59	59	50	85
*Mycoplasma pneumonia*	60	60	54	87
*Staphylococcal aureus*	54	54	67	92

Data are presented as percentages. Nucleotide and amino acid sequences of bacterial GAPDH registered with Kyoto Encyclopedia of Genes and Genomes (http://www.genome.jp/kegg/kegg_ja.html) were used, and identities and similarities were evaluated by Dr. Masayuki Fujino (the AIDS Research Center, National Institute of Infectious Diseases) using genetic information processing software (GENETYX-MAC ver. 18, GENETYX Corporation, Tokyo, Japan).
